# Fertility-Related Care for Gender and Sex Diverse Individuals: A Provider Needs-Assessment Survey

**DOI:** 10.1089/trgh.2016.0030

**Published:** 2016-10-01

**Authors:** Emilie K. Johnson, Diane Chen, Elisa J. Gordon, Ilina Rosoklija, Jane L. Holl, Courtney Finlayson

**Affiliations:** ^1^ Division of Urology, Ann & Robert H. Lurie Children's Hospital of Chicago, Chicago, Illinois.; ^2^ Department of Urology, Northwestern University Feinberg School of Medicine, Chicago, Illinois.; ^3^ Center for Healthcare Studies, Northwestern University Feinberg School of Medicine, Chicago, Illinois.; ^4^ Department of Child and Adolescent Psychiatry, Ann & Robert H. Lurie Children's Hospital of Chicago, Chicago, Illinois.; ^5^ Division of Adolescent Medicine, Ann & Robert H. Lurie Children's Hospital of Chicago, Chicago, Illinois.; ^6^ Division of Endocrinology, Ann & Robert H. Lurie Children's Hospital of Chicago, Chicago, Illinois.; ^7^ Department of Surgery, Northwestern University Feinberg School of Medicine, Chicago, Illinois.; ^8^ Department of Pediatrics, Northwestern University Feinberg School of Medicine, Chicago, Illinois.; ^9^ Department of Psychiatry and Behavioral Science, Northwestern University Feinberg School of Medicine, Chicago, Illinois.

**Keywords:** disorder of sex development, transgender persons, fertility, decision support techniques

## Abstract

Twelve providers (eight institutions) participating in a Gender and Sex Diversity Fertility Working Group completed a survey assessing their hospital's transgender, disorders/differences of sex development (DSD), and fertility healthcare teams. Participants also prioritized the development of a: (1) Provider Assessment Tool (questionnaire assessing knowledge/feelings about future fertility), or (2) decision aid (DA). Healthcare team presence by institution: transgender (6/8; 75%), DSD (75%), and fertility preservation (75%). Two-thirds of providers reported that the DA was most needed. Respondents recommended the DA to: help manage complex information, have a take-home reference, and provide neutral information. Other identified resource needs included: fertility specialists in clinic and financial resources.

## Introduction

The term “gender and sex diversity” encompasses the following: (1) transgender individuals for whom gender identity is incongruent with phenotypic sex, and (2) individuals with disorders/differences of sex development (DSD), for whom chromosomal, gonadal, or anatomic sex development is atypical.^1^ Although these two groups are largely distinct, there is overlap among the medical and mental health disciplines that care for both. In addition, many treatments (e.g., gender-affirming hormones, gonadectomy) currently offered to young transgender and DSD patients can affect future fertility.^2^ Medical decisions impacting the future fertility of transgender and DSD patients are complex, and often must be made in childhood, yet no clinical tools or guidelines exist to guide discussions about future fertility and fertility preservation (FP) with the families of gender and sex diverse individuals.

Similar to the parents of gender and sex diverse children, parents of pediatric cancer patients also face decisions related to potentially preserving their children's future fertility. Challenges in this setting include the following: (1) Necessity of parental proxy decision-making; (2) uncertainty regarding future assisted reproductive technologies, particularly related to maturation of immature reproductive tissue into usable adult eggs or sperm; and (3) cost of long-term tissue storage. For pediatric cancer patients, clinical guidelines regarding FP,^3,4^ and patient decision aids (DAs) are available to guide care.^5^

Parents contemplating the idea of preserving future fertility for their transgender child, or child with a DSD, face some distinct challenges compared with the pediatric cancer population. For example, families, patients, or providers may express hesitancy or unfamiliarity with utilizing gonads incongruent with gender identity to produce biological offspring. In addition, the fertility potential in many DSD conditions is still being defined. Despite the complexity of, and uncertainty about, pediatric FP options, and the unique issues facing gender and sex diverse individuals, no clinical tools exist to guide fertility-related care for these patients.

Our aim was to conduct a clinical needs-assessment of transgender and DSD care providers participating in a working group focused on fertility-related care. The needs-assessment was conducted to understand the perspectives and priorities of clinicians who care for transgender and DSD patients and to guide next steps for clinical tool development. We hypothesized that providers would recommend creating a clinical Provider Assessment Tool (vs. a DA) as the first step in clinical tool development.

## Materials and Methods

### Convening of the working group

The first multidisciplinary Gender and Sex Diversity Fertility Working Group was convened as a subgroup of the 2015 Oncofertility Consortium^®^ Meeting in Chicago.^6^ All Oncofertility Consortium Meeting attendees were invited to attend; participants were also invited based on previously expressed interest in the topic. Healthcare professionals from around North America with expertise in transgender, DSD, and fertility medicine met to share knowledge and experiences and to outline next steps for the field of fertility-related care for gender and sex diverse individuals. A needs-assessment survey was conducted to understand the current state of transgender, DSD, and FP teams at the institutions represented by working group members and to determine an initial priority for clinical tool development.

### Potential clinical tools

Ideas for the two potential clinical tools came from the Lurie Children's Gender and Sex Development Program (GSDP) Providers. GSDP providers who care for transgender and DSD patients have expressed difficulty with: (1) assessing families' knowledge, information needs, and desires about possible FP, and (2) counseling families about complex, often experimental, FP options. The GSDP clinic group expressed that a Provider Assessment Tool could potentially address the challenge with assessing knowledge and information needs and that a DA may assist with counseling about preservation options.

### Provider survey

Providers participating in the Gender and Sex Diversity Fertility Working Group completed a two-part self-administered survey (Supplementary Appendix 1). Section 1 focused on transgender, DSD, and FP healthcare team presence and composition. Section 2 asked respondents to rank order and detail reasons for recommending one of two potential clinical tools for development as follows: (1) a Provider Assessment Tool questionnaire assessing patient and family knowledge, thoughts, and feelings about fertility, or (2) a DA such as a pamphlet or website with information about fertility-related care and treatment options. Providers were also asked to suggest other clinical tools and resources needed to support fertility-related care for gender and sex diverse patients.

### Data analysis

Descriptive statistics were used to characterize survey responses. Reasons for recommending a Provider Assessment Tool or a DA were categorized and organized into themes. The Lurie Children's Institutional Review Board approved the study. Verbal consent was obtained from all participants.

## Results

### Participant characteristics and hospital healthcare teams

The Gender and Sex Diversity Fertility Working Group consisted of 14 participants (8 academic institutions, 7 areas of expertise), 12 of whom completed the survey (86% response rate). Freestanding children's hospitals, other pediatric hospitals, and adult institutions were all represented. Specialties and teams represented are detailed in Tables 1 and 2. Seventy-five percent of participating institutions (6/8) had a transgender healthcare team, 75% had a DSD team, and 75% had a FP team. Of institutions participating in the working group, 5/8 (63%) had all three (transgender, DSD, and FP) teams.

**Table 1.** **Participant Specialties and Teams**

	*N* (%)
Total institutions represented	8
Specialty	
Endocrinology	5 (4)
Gynecology	2 (1)
Urology	1 (8)
Psychology	1 (8)
Other—bioethics, oncology, reproductive endocrinology	3 (25)
Transgender Healthcare Team	
Yes—formal group	4 (50)
Yes—informal group	2 (25)
No/not sure	2 (25)
DSD Healthcare Team	
Yes—formal group	6 (75)
Yes—informal group	0 (0)
No/not sure	2 (25)
Fertility Preservation Healthcare Team	
Yes—formal group	3 (38)
Yes—informal group	3 (38)
No/not sure	2 (25)

**Table 2.** **Specialties Represented on Transgender, DSD, and Fertility Preservation Healthcare Teams**

	Transgender team (*N*=6), *N* (%)	DSD team (*N*=6), *N* (%)	Fertility team (*N*=6), *N* (%)
Adolescent medicine	4 (67)	—	—
Endocrinology	3 (50)	6 (100)	1 (17)
General Surgery	—	2 (33)	2 (33)
Urology	—	6 (100)	4 (67)
Gynecology	3 (50)	5 (83)	3 (50)
Psychology	6 (100)	4 (67)	1 (17)
Social work	5 (83)	3 (50)	1 (17)
Nursing	4 (67)	6 (100)	2 (33)
Genetics	—	5 (83)	—
Oncology	—	—	6 (100)
Other specialties	4^a^ (67)	1^b^ (17)	2^c^ (33)

^a^ Include medical ethics, clinic coordinator, child life, psychiatry, family medicine, plastic surgery, reproductive endocrinology, general pediatrics.

^b^ Include child life, neonatology.

^c^ Include research nurse, reproductive endocrinology.

DSD, disorders/differences of sex development.

### Clinical tool development preferences

Nine providers (75%) expressed a preference for developing either a Provider Assessment Tool or a DA. Three providers felt that both were equally important. Of providers with a preference, 6/9 (67%) prioritized the development of a patient DA and 3/9 (33%) preferred the Provider Assessment Tool. Reasons for preferring the DA included the following: (1) the DA could facilitate fertility discussions in the clinic, and (2) as an informational source, the DA could confer multiple benefits to patients and families. Specifically, respondents reported that the DA could help families manage a large volume of complex information, provide a take-home reference for later review, and serve as a neutral source of information about fertility options.

Participants who preferred the Provider Assessment Tool (*N*=3) believed that it could facilitate fertility-related discussions in clinic, similar to the DA. However, respondents also noted that the questionnaire-based assessment tool could help patients to clarify their values and help triage patients for referral to fertility specialists.

Other resource needs suggested included the following: (1) presence of a fertility specialist in transgender and DSD clinics, (2) a handout about what to expect if FP is desired, (3) connection to support groups to discuss fertility-related issues, (4) financial resources, (5) access to patient testimonials, and (6) development of visual aids to illustrate complex fertility-related concepts.

## Discussion

A multidisciplinary group of healthcare providers is necessary to provide fertility-related care for gender and sex diverse individuals. A sample of such providers recommended development of a patient DA to facilitate fertility-related care for transgender and DSD patients. The recommendation to create a patient DA about fertility was contrary to our expectation that providers would prefer a Provider Assessment Tool. Survey participants cited potential patient and family unfamiliarity with fertility as a topic, as well as management of a large volume of fertility-related information as potential benefits of the DA. Despite the potential for a Provider Assessment Tool to help focus initial clinic visits, the working group noted that topic of fertility should be introduced in person before use of a clinical tool.

Consideration of future fertility is relevant for both transgender and DSD patients, but the evidence base guiding fertility-related care for both groups is limited. For transgender individuals, medical interventions intended to alleviate gender dysphoria, including pubertal suppression and gender-affirming hormone therapy, may affect future fertility,^7^ but long-term effects are yet to be fully described. For DSD patients, fertility is more often impaired by the inherent gonadal developmental abnormalities or prophylactic gonadectomy performed due to malignancy risk. Fertility potential has been reported in the literature among individuals with selected DSD conditions, including Klinefelter^8–10^ and Turner Syndromes,^11,12^ but has been incompletely described for other DSD conditions. Relevant to both transgender and DSD individuals, pediatric FP options are still evolving. Many gender and sex diverse youth would require prepubertal gonadal cryopreservation to preserve future fertility. The technology to mature this tissue into adult forms of eggs or sperm is still in development, but fertility experts expect this option to be available in the future.^13–15^ Fertility-related decision-making for both DSD and transgender individuals occurs in the context of a high level of clinical uncertainty, highlighting the importance of developing clinical tools to guide care.

Developing a patient-centered DA about fertility is an important next step for our research group. DAs are now available for pediatric cancer patients,^5^ but similar tools are not available for gender and sex diverse individuals. We aim to develop a modular DA that could be adapted based on each patient's clinical situation and updated with emerging information and technologies. Previous investigators have noted the benefits of building in patient-level flexibility to clinical DAs.^16^ DAs have also been shown to improve care for many conditions by reducing decisional conflict, improving patient-clinician communication, and increasing satisfaction with decisions.^17^ Specific to fertility-related care for gender and sex diverse individuals, a patient-specific modular DA could help clinicians focus and structure discussions about this complicated evolving topic. Such a DA could also help to ensure that the entire multidisciplinary team is delivering a consistent message.

The institutions represented in our working group recognized the importance of a multidisciplinary team approach to transgender, DSD, and fertility-related care. However, the composition and organization of each of these teams varied by institution. In addition, the optimal model for interaction of these teams to provide fertility-related care to transgender and DSD patients is yet to be defined. It is encouraging that 63% of participating hospitals had either formal or informal teams covering all three care categories, suggesting that comprehensive fertility-related care for gender and sex diverse individuals could be accomplished at multiple sites nationwide.

The World Professional Association for Transgender Health Standards of Care^7^ and the 2006 DSD consensus statement^1^ both advocate for coordinated multidisciplinary care; we suggest that fertility-related care may be incorporated within these existing frameworks. A recommended minimum team composition includes: endocrinology, urology, pediatric surgery, fertility medicine, genetic counseling, and mental health, with other disciplines involved based on specific identified needs of each patient. Given that fertility-related care for gender and sex diverse individuals is a small and emerging field, telemedicine may facilitate this aspect of care for patients who otherwise may not have access. While all aspects of fertility-related care (e.g., surgical procedures) cannot be conducted remotely, telemedicine has an established role for certain aspects of care, including mental health,^18^ surgical consultation, and routine postoperative care.^19^ Thus, organizing and supporting teams to provide components of their clinical services remotely are recommended as the new healthcare field expands.

This study was limited by the sample size, and survey participants constituted a convenience sample with specialized interest and expertise in transgender, DSD, and fertility medicine. Given the small sample size, we were unable to stratify results by provider or hospital characteristics. However, we felt the survey was an important first step to query engaged providers about their needs, as there was no previous information available to guide our clinical tool development priorities. Another limitation was the format assessing preferred choice for clinical tool development—it was presented as a predefined dichotomous option. Although there was the opportunity for open-ended suggestions, there may be other clinical tools that would be more helpful that were not considered by the study team or survey participants. In addition, the survey did not request specific content recommendations for a Provider Assessment Tool or DA. To address this, we will be iteratively incorporating provider suggestions and feedback into the process of developing a DA about fertility for gender and sex diverse individuals. Finally, our investigation only represents the provider perspective, and we queried providers about both populations in aggregate. To mitigate this limitation, we are also currently obtaining and evaluating transgender and DSD patients and perspectives about how best to deliver fertility-related care.

## Conclusions

Fertility-related care for gender and sex diverse individuals is an emerging multidisciplinary field requiring multiple clinical tools. The first priority is to develop a patient DA, which will help families understand complex information about future fertility.

## Supplementary Material

Supplemental data

## Abbreviations Used

DA: decision aid
DSD: disorders/differences of sex development
FP: fertility preservation
GSDP: Gender and Sex Development Program
