# Supplementary material for: Fertility-Related Care for Gender and Sex Diverse Individuals: A Provider Needs-Assessment Survey
Source: Transgend Health. 2016 Oct 1;1(1):197–201. doi: 10.1089/trgh.2016.0030 (PMC5549540; doi:10.1089/trgh.2016.0030)
Supplement: Supplemental data [file Supp_App1.pdf]

Appendix 1. Survey Instrument

Northwestern Medicine | Ann & Robert H. Lurie Children's Hospital of Chicago

Provider Survey

Pediatric/Adolescent Gender and Sex Diversity Fertility Working Group

Questions about you and your team:

1. What is your primary area of focus?
   - ☐ Adolescent medicine
   - ☐ Endocrinology
   - ☐ General pediatrics
   - ☐ General surgery
   - ☐ Urology
   - ☐ Gynecology
   - ☐ Psychology
   - ☐ Social work
   - ☐ Nursing
   - ☐ Oncology
   - ☐ Another area_______________

2. Do you have a team that cares for transgender patients at your hospital?
   - ☐ Yes – this is a formally organized group
   - ☐ Yes – this is an informal group
   - ☐ No (skip to question 4)
   - ☐ Not sure (skip to question 4)

3. Which subspecialties are represented on your transgender health team? (check all that apply)
   - ☐ Adolescent medicine
   - ☐ Endocrinology
   - ☐ General pediatrics
   - ☐ General surgery
   - ☐ Urology
   - ☐ Gynecology
   - ☐ Psychology
   - ☐ Social work
   - ☐ Nursing
   - ☐ Other areas_________________

4. Do you have a team that cares for DSD patients at your hospital?
   - ☐ Yes – this is a formally organized group
   - ☐ Yes – this is an informal group
   - ☐ No (skip to question 6)
   - ☐ Not sure (skip to question 6)

5. Which subspecialties are represented on your DSD team? (check all that apply)
   - ☐ Endocrinology
   - ☐ General pediatrics
   - ☐ General surgery
   - ☐ Urology
   - ☐ Gynecology
   - ☐ Psychology
   - ☐ Social work
   - ☐ Nursing
   - ☐ Genetics
   - ☐ Other areas_________________

6. Do you have a fertility preservation team at your hospital?
   - ☐ Yes – this is a formally organized group
   - ☐ Yes – this is an informal group
   - ☐ No (skip to question 8)
   - ☐ Not sure (skip to question 8)

7. Which subspecialties are represented on your fertility preservation team? (check all that apply)
   - ☐ Endocrinology
   - ☐ General surgery

- ☐ Urology
- ☐ Gynecology
- ☐ Psychology
- ☐ Social work
- ☐ Nursing
- ☐ Oncology
- ☐ Other areas____________________

Questions about what is needed:

We've identified 2 ideas for clinical tools that could help transgender and DSD patients and their providers. We would like your feedback as to which tool type you think we should work on developing:

A. A Patient/Family Questionnaire: This could also be called a *Provider Assessment Tool.* The questionnaire would be filled out by the patient/parent before or during their clinic visit. It would assess knowledge, thoughts, and feelings about fertility. The responses would then help the doctor or nurse guide the fertility-related discussion during the clinic visit.

B. A Patient Decision Aid: This aid could be a pamphlet or website with information about DSD/transgender patients. It would provide the patient/family with neutral information about their fertility options, including pros and cons, to help them make a decision about fertility-related treatment. This would be provided sometime after initial discussions about fertility in clinic.

1. Which of the ideas described above do you think is needed most?
   - ☐ A – Provider Assessment Tool
   - ☐ B – Patient Decision Aid

2. Consider the tool you selected. How could this tool help you?

____

____

____

3. Consider the tool you selected. How could this tool help your patients and families?

____

____

____

4. In your opinion, what other types of clinical tools might be useful for transgender and/or DSD patients who are considering fertility-related issues?

____

____

____
